# Ethyl 1-aminona­phtho[2,1-*b*]furan-2-carboxyl­ate

**DOI:** 10.1107/S1600536812025342

**Published:** 2012-06-16

**Authors:** K. S. Madan, E. Shruthi, A. M. Sagar, M. M. M Abdoh, V. P. Vaidya, N. K. Lokanath

**Affiliations:** a Department of Studies in Physics, Manasagangotri, University of Mysore, Mysore 570 006, India; b Department of Chemistry, Kuvempu University, Shankaraghatta 577 451, India; c Department of Physics, Faculty of Science, An Najah National University, Nabtus, West Bank, Palestinian Territories

## Abstract

In the title compound, C_15_H_13_NO_3_, there is intra­molecular N—H⋯O hydrogen bond between the amino group and the ester carbonyl O atom and the dihedral angle between the aromatic ring and the ester group is 2.05 (15)°. In the crystal, mol­ecules are connected by N—H⋯O hydrogen bonds into chains parallel to [010]. In addition there are short C—H⋯O inter­actions and π–π stacking inter­actions with a distance of 3.555 (2) Å between the centroids of the furan and benzene rings.

## Related literature

For bioactivity of naphtho­furan compounds, see: Nagaraja *et al.* (2006 ▶); Mahadevan *et al.* (2005 ▶). For similar structures, see: Shruthi *et al.* (2012 ▶). For the synthesis of the title compound, see: Veena *et al.* (2011 ▶)
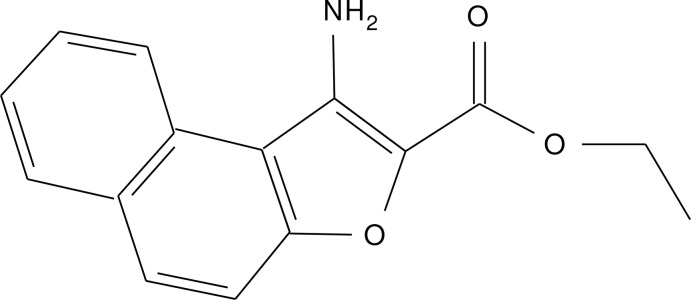


## Experimental

### 

#### Crystal data

C_15_H_13_NO_3_
*M* *_r_* = 255.26
Orthorhombic, 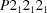

*a* = 6.2217 (5) Å
*b* = 8.3795 (6) Å
*c* = 23.4692 (17) Å
*V* = 1223.56 (16) Å^3^
*Z* = 4
Mo *K*α radiation
μ = 0.10 mm^−1^
*T* = 103 K
0.28 × 0.22 × 0.22 mm

#### Data collection

Oxford Diffraction Xcalibur Eos diffractometer
18171 measured reflections
1288 independent reflections
1093 reflections with *I* > 2σ(*I*)
*R*_int_ = 0.064

#### Refinement

*R*[*F* ^2^ > 2σ(*F* ^2^)] = 0.045
*wR*(*F* ^2^) = 0.117
*S* = 1.03
1288 reflections
173 parameters
H-atom parameters constrained
Δρ_max_ = 0.31 e Å^−3^
Δρ_min_ = −0.36 e Å^−3^

### 

Data collection: *CrysAlis PRO* (Oxford Diffraction, 2010 ▶); cell refinement: *CrysAlis PRO*; data reduction: *CrysAlis PRO*; program(s) used to solve structure: *SHELXS97* (Sheldrick, 2008 ▶); program(s) used to refine structure: *SHELXL97* (Sheldrick, 2008 ▶); molecular graphics: *PLATON* (Spek, 2009 ▶); software used to prepare material for publication: *PLATON*.

## Supplementary Material

Crystal structure: contains datablock(s) global, I. DOI: 10.1107/S1600536812025342/gk2494sup1.cif

Structure factors: contains datablock(s) I. DOI: 10.1107/S1600536812025342/gk2494Isup2.hkl

Supplementary material file. DOI: 10.1107/S1600536812025342/gk2494Isup3.cml

Additional supplementary materials:  crystallographic information; 3D view; checkCIF report

## Figures and Tables

**Table 1:** Hydrogen-bond geometry (Å, °)

*D*—H⋯*A*	*D*—H	H⋯*A*	*D*⋯*A*	*D*—H⋯*A*
N4—H4*A*⋯O2	0.86	2.30	2.872 (4)	124
N4—H4*B*⋯O2^i^	0.86	2.29	3.027 (4)	144
C9—H9⋯O2^i^	0.93	2.50	3.308 (4)	146

Symmetry code: (i) 
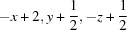
.

**Fig. 1.**
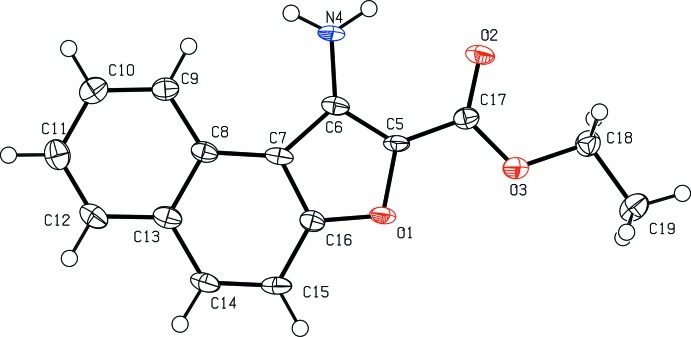
ORTEP diagram of the title compound with 50% probability ellipsoids.

**Fig. 2.**
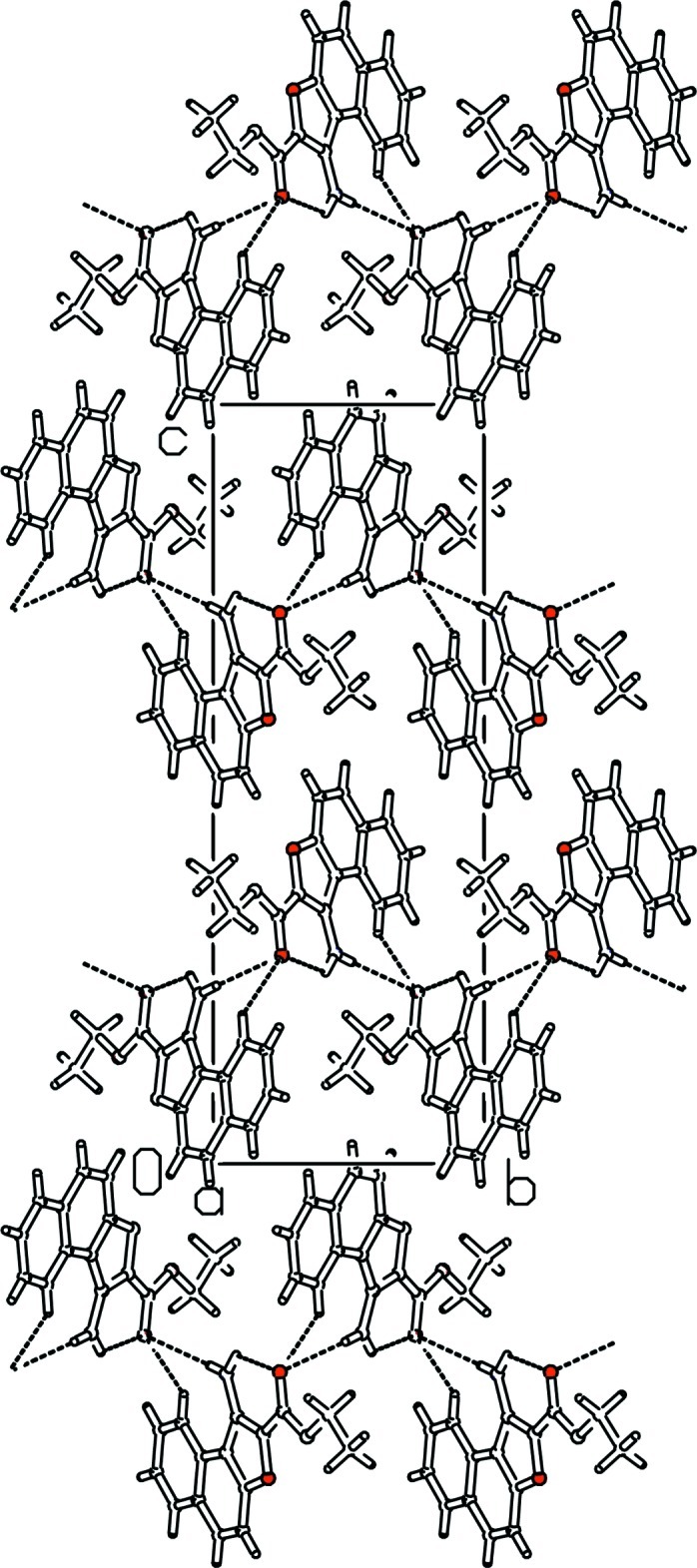
Packing diagram of the title compound viewed along the crystallographic a axis. N—H···O hydrogen bonds are indicated by dashed lines.

## supplementary crystallographic information

### Comment

Various derivatives of naphtho[2,1-b]furan synthesized in our laboratory have been shown to possess broad spectrum of pharmacological and biological activities (Nagaraja *et al.*, 2006).

The title compound was synthesized as an intermediate in the synthesis of aza heterocyclic derivative of naphtho[2,1-b]furan. The synthesis of the title compound was reported by Veena *et al.* (2011).

The ORTEP drawing of the title molecule is shown in Fig. 1. The naphthofuran system is basically planar and its geometry is similar to ethyl naphtho[2,1- b]furan-2-carboxylate (Shruthi *et al.*, 2012). The dihedral angle between aromatic ring and the ester group is 2.05 (15)°.

The molecules are connected by N-H···O interactions into chains along the *b* axis (Fig. 2). There are *π*-*π* stacking interactions between molecules related by unit translation along the *a* axis with the distance of 3.555 (2) Å between centroids of the furan and benzene rings.

### Experimental

Ethyl 3-aminonaphtho[2,1-*b*]furan-2-carboxylate was synthesized as per the procedure reported in the literature (Veena *et al.*, 2011). The final product was obtained by recrystallization using aqueous ethanol as a solvent. Slow evaporation method yielded crystals.

### Refinement

In the absence of significant anomalous dispersion effects Friedel pairs have been merged. All the hydrogen atoms of the compound are fixed geometrically (N-H = 0.86 and C-H= 0.93-0.97 Å) and allowed to ride on their parent atoms.

### Crystal data

**Table:**

C_15_H_13_NO_3_	*F*(000) = 536
*M**_r_* = 255.26	*D*_x_ = 1.386 Mg m^−^^3^
Orthorhombic, *P*2_1_2_1_2_1_	Mo *K*α radiation, λ = 0.71073 Å
Hall symbol: P 2ac 2ab	Cell parameters from 1288 reflections
*a* = 6.2217 (5) Å	θ = 1.7–25.0°
*b* = 8.3795 (6) Å	µ = 0.10 mm^−^^1^
*c* = 23.4692 (17) Å	*T* = 103 K
*V* = 1223.56 (16) Å^3^	Block, colorless
*Z* = 4	0.28 × 0.22 × 0.22 mm

### Data collection

**Table:**

Oxford Diffraction Xcalibur Eos diffractometer	1093 reflections with *I* > 2σ(*I*)
Radiation source: fine-focus sealed tube	*R*_int_ = 0.064
Graphite monochromator	θ_max_ = 25.0°, θ_min_ = 1.7°
Detector resolution: 16.0839 pixels mm^-1^	*h* = −7→7
ω scans	*k* = −9→9
18171 measured reflections	*l* = −27→26
1288 independent reflections	

### Refinement

**Table:**

Refinement on *F*^2^	Primary atom site location: structure-invariant direct methods
Least-squares matrix: full	Secondary atom site location: difference Fourier map
*R*[*F*^2^ > 2σ(*F*^2^)] = 0.045	Hydrogen site location: inferred from neighbouring sites
*wR*(*F*^2^) = 0.117	H-atom parameters constrained
*S* = 1.03	*w* = 1/[σ^2^(*F*_o_^2^) + (0.0749*P*)^2^ + 0.4783*P*] where *P* = (*F*_o_^2^ + 2*F*_c_^2^)/3
1288 reflections	(Δ/σ)_max_ < 0.001
173 parameters	Δρ_max_ = 0.31 e Å^−^^3^
0 restraints	Δρ_min_ = −0.36 e Å^−^^3^

### Special details

**Table:**

Geometry. Bond distances, angles *etc*. have been calculated using the rounded fractional coordinates. All su's are estimated from the variances of the (full) variance-covariance matrix. The cell e.s.d.'s are taken into account in the estimation of distances, angles and torsion angles
Refinement. Refinement on *F*^2^ for ALL reflections except those flagged by the user for potential systematic errors. Weighted *R*-factors *wR* and all goodnesses of fit *S* are based on *F*^2^, conventional *R*-factors *R* are based on *F*, with *F* set to zero for negative *F*^2^. The observed criterion of *F*^2^ > σ(*F*^2^) is used only for calculating -*R*-factor-obs *etc*. and is not relevant to the choice of reflections for refinement. *R*-factors based on *F*^2^ are statistically about twice as large as those based on *F*, and *R*-factors based on ALL data will be even larger.

### Fractional atomic coordinates and isotropic or equivalent isotropic displacement parameters (Å^2^)

**Table:**

	*x*	*y*	*z*	*U*_iso_*/*U*_eq_	
O1	0.9439 (3)	0.7967 (3)	0.08591 (8)	0.0215 (7)	
O2	0.7201 (4)	0.7539 (3)	0.22562 (9)	0.0245 (7)	
O3	0.6156 (4)	0.6531 (3)	0.14062 (9)	0.0243 (7)	
N4	1.0980 (4)	0.9507 (3)	0.22348 (11)	0.0233 (8)	
C5	0.9186 (5)	0.8149 (4)	0.14469 (12)	0.0196 (9)	
C6	1.0771 (5)	0.9106 (4)	0.16666 (13)	0.0200 (10)	
C7	1.2157 (5)	0.9524 (4)	0.11969 (12)	0.0194 (9)	
C8	1.4090 (5)	1.0413 (4)	0.11392 (13)	0.0199 (9)	
C9	1.5215 (5)	1.1151 (4)	0.15900 (13)	0.0220 (9)	
C10	1.7085 (5)	1.1961 (4)	0.14924 (14)	0.0258 (10)	
C11	1.7922 (6)	1.2091 (4)	0.09382 (14)	0.0276 (10)	
C12	1.6865 (6)	1.1397 (4)	0.04934 (14)	0.0263 (11)	
C13	1.4948 (6)	1.0545 (4)	0.05737 (13)	0.0226 (9)	
C14	1.3886 (6)	0.9777 (4)	0.01060 (13)	0.0252 (10)	
C15	1.2054 (6)	0.8914 (4)	0.01695 (12)	0.0241 (10)	
C16	1.1224 (5)	0.8797 (4)	0.07259 (13)	0.0201 (9)	
C17	0.7458 (5)	0.7406 (4)	0.17366 (13)	0.0197 (10)	
C18	0.4392 (5)	0.5720 (4)	0.16960 (14)	0.0249 (10)	
C19	0.3046 (6)	0.4943 (4)	0.12417 (15)	0.0302 (11)	
H4A	1.00740	0.91480	0.24800	0.0280*	
H4B	1.20160	1.01150	0.23430	0.0280*	
H9	1.46750	1.10840	0.19590	0.0260*	
H10	1.78100	1.24320	0.17960	0.0310*	
H11	1.91940	1.26470	0.08750	0.0330*	
H12	1.74280	1.14920	0.01280	0.0320*	
H14	1.44760	0.98740	−0.02560	0.0300*	
H15	1.13830	0.84250	−0.01390	0.0290*	
H18A	0.35390	0.64800	0.19110	0.0300*	
H18B	0.49460	0.49230	0.19570	0.0300*	
H19A	0.25390	0.57410	0.09810	0.0450*	
H19B	0.18410	0.44170	0.14150	0.0450*	
H19C	0.38990	0.41750	0.10390	0.0450*	

### Atomic displacement parameters (Å^2^)

**Table:**

	*U*^11^	*U*^22^	*U*^33^	*U*^12^	*U*^13^	*U*^23^
O1	0.0281 (12)	0.0267 (12)	0.0098 (10)	−0.0011 (11)	0.0003 (9)	−0.0002 (9)
O2	0.0339 (13)	0.0273 (13)	0.0122 (11)	0.0006 (12)	0.0006 (9)	0.0017 (9)
O3	0.0282 (12)	0.0274 (12)	0.0173 (11)	−0.0038 (11)	−0.0004 (9)	−0.0010 (9)
N4	0.0301 (15)	0.0319 (15)	0.0080 (12)	−0.0032 (15)	−0.0007 (11)	−0.0010 (11)
C5	0.0276 (17)	0.0217 (16)	0.0094 (13)	−0.0001 (16)	−0.0013 (13)	−0.0003 (12)
C6	0.0288 (18)	0.0185 (17)	0.0126 (15)	0.0043 (15)	0.0007 (13)	0.0009 (12)
C7	0.0282 (17)	0.0197 (16)	0.0104 (14)	0.0061 (15)	0.0012 (14)	−0.0007 (12)
C8	0.0270 (17)	0.0173 (16)	0.0153 (15)	0.0042 (16)	0.0013 (14)	0.0020 (12)
C9	0.0255 (17)	0.0211 (16)	0.0193 (16)	0.0032 (16)	−0.0019 (14)	−0.0002 (13)
C10	0.0281 (18)	0.0224 (18)	0.0268 (17)	0.0030 (17)	−0.0058 (14)	0.0010 (15)
C11	0.0261 (18)	0.0242 (18)	0.0324 (18)	0.0009 (17)	0.0018 (15)	0.0057 (15)
C12	0.0325 (19)	0.0256 (19)	0.0207 (17)	0.0016 (16)	0.0074 (15)	0.0033 (14)
C13	0.0289 (17)	0.0212 (17)	0.0177 (15)	0.0065 (16)	0.0020 (14)	0.0009 (13)
C14	0.038 (2)	0.0237 (18)	0.0138 (15)	0.0006 (18)	0.0060 (14)	−0.0007 (13)
C15	0.0369 (19)	0.0236 (18)	0.0119 (15)	0.0025 (17)	−0.0006 (14)	−0.0037 (13)
C16	0.0236 (16)	0.0209 (16)	0.0157 (15)	0.0016 (15)	0.0002 (13)	0.0022 (13)
C17	0.0237 (17)	0.0177 (17)	0.0177 (16)	0.0021 (15)	−0.0026 (13)	0.0007 (13)
C18	0.0269 (18)	0.0228 (18)	0.0249 (17)	−0.0034 (16)	−0.0020 (14)	0.0014 (14)
C19	0.033 (2)	0.0276 (19)	0.0300 (19)	−0.0028 (17)	−0.0091 (16)	−0.0003 (15)

### Geometric parameters (Å, º)

**Table:**

O1—C5	1.397 (3)	C11—C12	1.364 (5)
O1—C16	1.347 (4)	C12—C13	1.403 (5)
O2—C17	1.235 (4)	C13—C14	1.434 (5)
O3—C17	1.340 (4)	C14—C15	1.358 (5)
O3—C18	1.459 (4)	C15—C16	1.408 (4)
N4—C6	1.381 (4)	C18—C19	1.504 (5)
N4—H4A	0.8600	C9—H9	0.9300
N4—H4B	0.8600	C10—H10	0.9300
C5—C17	1.416 (4)	C11—H11	0.9300
C5—C6	1.372 (4)	C12—H12	0.9300
C6—C7	1.443 (4)	C14—H14	0.9300
C7—C16	1.389 (4)	C15—H15	0.9300
C7—C8	1.421 (4)	C18—H18A	0.9700
C8—C9	1.411 (4)	C18—H18B	0.9700
C8—C13	1.435 (4)	C19—H19A	0.9600
C9—C10	1.366 (4)	C19—H19B	0.9600
C10—C11	1.405 (5)	C19—H19C	0.9600
			
C5—O1—C16	105.4 (2)	O1—C16—C15	123.6 (3)
C17—O3—C18	116.1 (2)	O2—C17—O3	122.9 (3)
H4A—N4—H4B	120.00	O2—C17—C5	122.2 (3)
C6—N4—H4B	120.00	O3—C17—C5	114.9 (3)
C6—N4—H4A	120.00	O3—C18—C19	106.9 (3)
C6—C5—C17	128.5 (3)	C8—C9—H9	119.00
O1—C5—C17	120.8 (3)	C10—C9—H9	120.00
O1—C5—C6	110.7 (3)	C9—C10—H10	120.00
N4—C6—C5	125.0 (3)	C11—C10—H10	120.00
N4—C6—C7	128.5 (3)	C10—C11—H11	120.00
C5—C6—C7	106.5 (3)	C12—C11—H11	120.00
C8—C7—C16	120.5 (3)	C11—C12—H12	119.00
C6—C7—C8	134.9 (3)	C13—C12—H12	119.00
C6—C7—C16	104.6 (3)	C13—C14—H14	119.00
C7—C8—C9	125.3 (3)	C15—C14—H14	119.00
C7—C8—C13	116.3 (3)	C14—C15—H15	122.00
C9—C8—C13	118.4 (3)	C16—C15—H15	122.00
C8—C9—C10	121.0 (3)	O3—C18—H18A	110.00
C9—C10—C11	120.6 (3)	O3—C18—H18B	110.00
C10—C11—C12	119.8 (3)	C19—C18—H18A	110.00
C11—C12—C13	121.6 (3)	C19—C18—H18B	110.00
C8—C13—C14	120.1 (3)	H18A—C18—H18B	109.00
C8—C13—C12	118.7 (3)	C18—C19—H19A	109.00
C12—C13—C14	121.2 (3)	C18—C19—H19B	109.00
C13—C14—C15	122.8 (3)	C18—C19—H19C	109.00
C14—C15—C16	116.5 (3)	H19A—C19—H19B	109.00
C7—C16—C15	123.7 (3)	H19A—C19—H19C	109.00
O1—C16—C7	112.7 (3)	H19B—C19—H19C	109.00
			
C16—O1—C5—C6	1.3 (4)	C16—C7—C8—C13	1.9 (5)
C16—O1—C5—C17	−179.2 (3)	C6—C7—C16—O1	−0.8 (4)
C5—O1—C16—C7	−0.3 (4)	C6—C7—C16—C15	179.3 (3)
C5—O1—C16—C15	179.7 (3)	C8—C7—C16—O1	178.2 (3)
C18—O3—C17—O2	−0.3 (4)	C8—C7—C16—C15	−1.8 (5)
C18—O3—C17—C5	178.6 (3)	C7—C8—C9—C10	178.9 (3)
C17—O3—C18—C19	175.1 (3)	C13—C8—C9—C10	−0.5 (5)
O1—C5—C6—N4	−179.5 (3)	C7—C8—C13—C12	−179.4 (3)
O1—C5—C6—C7	−1.8 (4)	C7—C8—C13—C14	−1.2 (5)
C17—C5—C6—N4	1.1 (6)	C9—C8—C13—C12	0.1 (5)
C17—C5—C6—C7	178.8 (3)	C9—C8—C13—C14	178.3 (3)
O1—C5—C17—O2	179.6 (3)	C8—C9—C10—C11	0.6 (5)
O1—C5—C17—O3	0.7 (4)	C9—C10—C11—C12	−0.3 (5)
C6—C5—C17—O2	−1.0 (6)	C10—C11—C12—C13	−0.1 (5)
C6—C5—C17—O3	−179.9 (3)	C11—C12—C13—C8	0.2 (5)
N4—C6—C7—C8	0.4 (6)	C11—C12—C13—C14	−178.0 (3)
N4—C6—C7—C16	179.1 (3)	C8—C13—C14—C15	0.4 (5)
C5—C6—C7—C8	−177.2 (4)	C12—C13—C14—C15	178.6 (3)
C5—C6—C7—C16	1.5 (4)	C13—C14—C15—C16	−0.2 (5)
C6—C7—C8—C9	1.0 (6)	C14—C15—C16—O1	−179.1 (3)
C6—C7—C8—C13	−179.6 (4)	C14—C15—C16—C7	0.9 (5)
C16—C7—C8—C9	−177.6 (3)		

### Hydrogen-bond geometry (Å, º)

**Table:**

*D*—H···*A*	*D*—H	H···*A*	*D*···*A*	*D*—H···*A*
N4—H4*A*···O2	0.86	2.30	2.872 (4)	124
N4—H4*B*···O2^i^	0.86	2.29	3.027 (4)	144
C9—H9···O2^i^	0.93	2.50	3.308 (4)	146

Symmetry code: (i) −*x*+2, *y*+1/2, −*z*+1/2.
